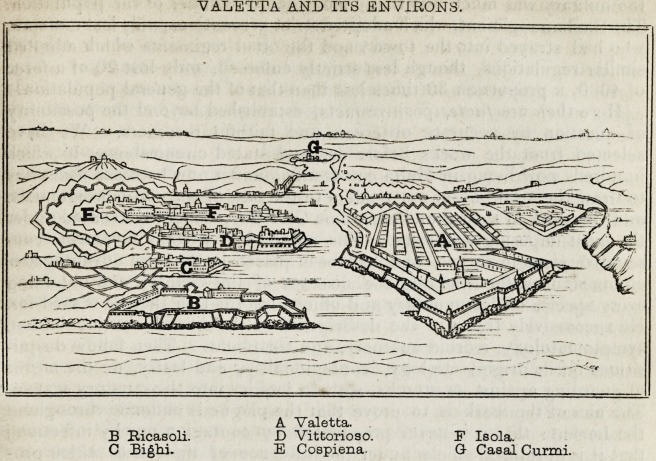# On the Oriental Plague, from Materials Collected in Alexandria, Cairo, Smyrna, and Constantinople, during the Years 1833-38

**Published:** 1843-10

**Authors:** 


BRITISH AND FOREIGN

### MEDICAL REVIEW,

FOR OCTOBER, 1843.

### PART FIRST.

### Analytical and Critical Reviews.

### Art. I.

1. *De la Peste Orientale, d'après les matériaux recueillis à Alexandrie, au Caire, à Smyrne, et à Constantinople, pendant les années* 1833-38. Par A. F. Bulard, de Méru ; chargé de mission par le gouvernement français pour l'observation de la Peste dans toutes les localités de l'Empire Ottoman ; inspecteur du service de la marine Egyptienne ; et médecin en chef de l'hôpital militaire du Caire, etc. etc.—*Paris*, 1839. 8vo, pp. 422.

*On the Oriental Plague, from materials collected in Alexandria, Cairo, Smyrna, and Constantinople, during the years* 1833-38. By A. F. Bulard, of Méru ; Commissioner appointed by the French government for the observation of the Plague throughout the Ottoman Empire ; Inspector of the Egyptian Navy ; and chief Physician of the Military Hospital of Cairo, &c. &c.—*Paris*, 1839.

2. *De la Peste observée en Egypte. Recherches et Considérations sur cette Maladie.* Par Clot-Bey.—*Paris*, 1840. 8vo.

*Researches and Considerations on the Plague as observed in Egypt.* By Clot-Bey.—*Paris*, 1840.

3. *Notes and Observations on the Ionian Islands and Malta, with some Remarks on Constantinople and Turkey, and on the System of Quarantine as at present conducted.* By John Davy, m.d. f.r.ss.l. & e. Inspector-general of Army Hospitals.—*London*, 1842. 2 vols. 8vo, pp. 436, 478.

4. *Rapport addressé à S. E. le Ministre de l'Agriculture et du Commerce sur des Modifications à apporter aux Réglements sanitaires.* Par M. de Ségur-Dupeyron, Secrétaire du Conseil supérieur de Santé, etc. —*Paris*, 1839. 8vo, pp. 147.

*Report addressed to his Excellency the Minister of Agriculture and Commerce, on Modifications of the Sanatory Regulations.* By M. de Ségur-Dupeyron, Secretary of the Superior Council of Health, &c.—*Paris*, 1839.

5. *Elements of Medicine.* Vol. II.—*Morbid Poisons.* By ROBERT WILLIAMS, M.D., President of the Royal Medical and Chirurgical Society, and Senior-Physician of St. Thomas's Hospital, &c.—*London*, 1841. 8vo, pp. 686.

6. *Rapporto officiale fatto al sopra-intendente alla Quarantina di alcuni casi di Peste.* Scritto da LUIGI GRAVAGNA, M.D., medico principale di Sanita.—*Malta*, 1841. 8vo, p. 12.

*Official Report of certain cases of Plague made to the Superintendent of Quarantine.* By L. GRAVAGNA, M.D., Principal Physician of the Quarantine Establishment.

7. *Lettre première au sujet des Accidents de Peste survenus tant au Lazaret de Koulély qu'a l'île de Proti, &c.* Par ANTOINE PEZZONI, M.D., Membre du Conseil supérieur de Santé Ottoman, etc.—*Constantinople*, 1841. 8vo, pp. 15.

*First Letter on Cases of Plague which occurred in the Lazaretto of Koulély and the Isle of Proti, &c.* By A. PEZZONI, M.D., Member of the Turkish Superior Council of Health, &c.—*Constantinople*, 1841.

8. *The Quarantine Laws, their abuses and inconsistencies; a Letter addressed to Sir John C. Hobhouse, M.P., President of the Board of Control.* By ARTHUR T. HOLROYD, Esq.—*London*, 1839. 8vo, pp. 65.

9. *Report from the Select Committee of the House of Commons on the Contagion of Plague.*—*London*, 1819. Folio, pp. 102.

IF any proof were wanting of the unsettled state of opinion among the medical profession on the subject of the contagious nature of plague, it would be fully supplied by a glance at the works we have just enumerated. We have here men of high reputation and evident ability, who have the most ample opportunities for observation and inquiry, arriving at the most opposite conclusions, and entertaining the most contradictory opinions. One asserts positively that the plague is truly a contagious disease, and may be propagated either by direct contact with diseased persons, or indirectly through the medium of various articles which have been in contact with such persons; and that the protection of the public health requires the establishment of quarantine regulations for the purpose of preventing the importation of the disease from diseased into healthy countries, by means of persons or goods. We have another equally confident that the plague is a purely endemic or epidemic disease, having a local origin from malaria or other causes in certain districts, and that it cannot be communicated from a diseased to a healthy person; consequently, that quarantine regulations, without being of the slightest service in preventing its extension, are expensive and vexatious impediments to commerce, causing immense loss of interest and time, injury to merchandise, fluctuation of markets, and great personal inconvenience.

Such a contrariety of opinion upon one of the few questions in which our profession is consulted by the legislative power, is peculiarly unsatisfactory; and as recent parliamentary inquiries would make it appear that our government, the head of the first commercial system in the world, will take that share in its settlement which the magnitude of our interests in the Mediterranean demands, we shall endeavour to lay before our readers the real state of our knowledge on the subject, drawn from the works before us and personal observation in the Mediterranean. We do not purpose to give a critical analysis of any of these works, as in the present inquiry we have little to do with the detailed account of the history of the disease,—the various speculations as to its remote and predisposing causes,—its coexistence with other diseases,—its pathology, symptoms, diagnosis, or treatment. These may be found in any standard essay on plague, and do not bear so directly on the quarantine question; we therefore confine ourselves to the inquiries: How is the plague propagated? If by contagion, what are the best means of arresting its progress? How far do the present quarantine establishments fulfil these objects; in what respects are their regulations at variance with scientific observation; and how may they be regulated with full regard to the safety of the public, and the smallest restrictions upon commerce?

I. In proceeding with the inquiry, *How is the plague propagated?* let us determine what are the tests by which it may be proved that any given disease may be received by a healthy person in consequence of direct communication with the sick, and then ascertain how far the laws of plague are in accordance with such tests. In the first place, then, it is self-evident that when a disease can be communicated by inoculation, that disease is contagious: again, there is the strongest reason to suppose a disease contagious when it arises in a place previously healthy, immediately after the arrival of a diseased person or persons, or articles of merchandize from infected places, especially when those first attacked are those who have been exposed to direct communication with the diseased persons or infected merchandise: thirdly, a similar argument is well founded when those persons whose duties bring them into more direct communication with the sick, are affected by the disease in a greater proportion than the rest of the population, and, *a fortiori*, when the proportion of the former varies with their more or less continued communication with the sick: lastly, when seclusion and separation from diseased persons, and from all objects which have been brought into contact with such persons, have an evident influence in protecting those secluded from a prevailing disease, other things being equal, the conclusion in favour of contagion is very strongly grounded.

With regard to the effects of inoculation the evidence is contradictory. Clot-Bey inoculated himself with blood and pus from a plague patient without any result, while Dr. White after two unsuccessful attempts, caught the plague after the third, and died in three days. Dr. Valli in 1803, died after a similar experiment. Deggio, a Russian army-surgeon, inoculated himself "at Bucharest in 1773, and took the plague on the fourth day, but survived." (Williams, p. 286.) In 1818-19 Dr. Sola inoculated fourteen condemned Spanish deserters at Tangier, seven of whom are said to have taken the plague. (Clot-Bey, p. 349.) M. Bulard inoculated four persons with blood, pus from the buboes, and serosity from the phlyctena of the carbuncle of plague patients. One only took the disease, and recovered, and he was exposed to the infection of a diseased neighbourhood. (Bulard, 141-4.) Dr. Grassi, Protomedico di Sanità at Alexandria, has recorded the case of a physician who pricked himself on the ring finger of the left hand in opening the dead body of a plague patient. Four days afterwards he had cold chills, vomiting, headach, and lumbar pains, which continued during the next day, when he noticed a small phlyctena on the finger, and a painful swelling of the corresponding axilla. On the following day the general symptoms increased to delirium, and the finger and axilla had become the seats of plague carbuncle and bubo. He ultimately recovered. (Il Filocamo, 25 Aprile, 1842.) This we believe to be the sum of veritable evidence on the results of inoculation, as the experiments on animals are valueless, until it can be shown that the plague is not a disease peculiar to the human subject. Further inquiry is necessary on this point, because the noncontagionist may urge that the above experiments were made during a prevalence or epidemic of plague, and therefore it is uncertain where the disease arose independently of inoculation. This argument, however, could scarcely be tenable, where, as in Dr. Grassi's case, a carbuncle formed at the seat of puncture.

If we examine the evidence of contagion as derived from the importation of plague into a place previously healthy, by the arrival of persons from infected districts, we shall find an immense number of cases recorded, (a very good summary of which is given in the work of M. Ségur-Dupeyron,) in which plague has followed the arrival of ships having plague on board. We, however, do not insist on these, as the greater part occurred in places subject to occasional visitations of the pestilence, and more especially as we have ample means of following up the inquiry where no such objection can be urged, namely, in the island of Malta, a dry rock of limestone, free from any source of malaria which could be conceived possible to give origin to the disease. In 1813 this island had been free from plague for 130 years, when a ship arrived on the 29th of March from Alexandria, where the plague was raging at the time of her departure, and she had lost two men by plague during the passage. Three days after her arrival in Malta the master also died, the ship lying in the harbour close to the city of Valetta. The inhabitants became very uneasy and some merchants remonstrated against the vessel remaining in the harbour, and Sir A. B. Faulkner, at that time physician to the forces in the island, addressed an official letter to the Commander-in-chief, recommending the immediate removal of the vessel. This was not attended to, and on the 16th of April, *six days after this admonitory letter was written*, the first case of plague occurred in Valetta. (Evidence of Sir A. B. Faulkner, Parliamentary Report, pp. 46.) The quarantine regulations had been very lax; some new linen was found in the house of Borg, where the first case occurred; this linen corresponded with that of a missing bale from the ship, and Borg died crying, "Oh the linen, the linen." Borg and two others of his family were the first victims; one died on the 19th of April, the two others on the 2d of May. The next house attacked was that of a schoolmistress, an intimate friend of Borg, who had attended him during his illness, and the progress of the contagion was also clearly traced through some of her scholars. After this, in the words of Sir A. Faulkner, "The foci of contagion became so rapidly multiplied, that it appeared to me impossible to carry the investigation in a direct line further in that populous city; but I am in possession of documents furnished to me by one of the captains of the Lazaretto himself, a man of strict integrity, and many years employed in that official situation, showing that the contagion made its way in a direct line from Valetta into most of the infected capals or villages." (Parliamentary Report, p.47.)

Here then we have as striking an example as can possibly be conceived of importation of disease into a healthy community, those being first affected who communicated with the disease, and the course of the contagion being afterwards traceable. From the pamphlets of M. Pezzoni and Dr. Gravagna, we draw other undoubted proofs of importation. The letter of M. Pezzoni was addressed to Dr. Davy, and that gentleman after having written a long chapter, in which he has strongly supported the case of the non-contagionists, allows in a foot-note, (Davy, p. 133,) that it "carried conviction to my mind, previously in doubt on the question." The facts are shortly these: On June 8, 1841, a merchant vessel arrived at Constantinople from Alexandria, with some cases of plague among the passengers and crew. Constantinople and the neighbourhood had been free from plague for three years previously. A lazaretto guardian in perfect health was sent on board and assisted in landing the patients. He was taken ill on the 13th and died on the 15th with bubo and every symptom of plague. A porter also on the 22d was found to have had symptoms of plague for two days, and a very large bubo in the left groin followed. He was conveyed to the pest-house and recovered. Two other employées of the lazaret died on the 15th and 17th of July. Here then, we have a city of 800,000 inhabitants, free from epidemic influence; healthy persons are placed in contact with plague patients, four take the disease and three die. The disease is confined within the walls of the lazaret, no one but those brought into connexion with infected persons suffer; and the city has since remained healthy.

Similar facts are related by Dr. Gravagna. On the 26th of May, 1841, a vessel arrived in Malta from Alexandria, with plague on board among the sailors and pilgrims. The whole of the crew, &c., of the vessel, with guardians and two boatmen who had communicated with the pilgrims were segregated, and every precaution taken to prevent the spread of the disease. Cases appeared among the crew on the 4th and 6th of June, making in all ten cases, one showing itself during the voyage, and nine others in lazaret. One of the Maltese boatmen communicated with the vessel on the 28th of May, and on the 6th of June he was attacked by plague, with carbuncle and bubo, and died on the 10th. The other boatman was not attacked, nor were the guardians. Here, then, we see that the only man of a large population attacked by the disease, was one who was brought into contact with the diseased. He was confined in the lazaret, and the disease did not extend beyond the walls. Dr. Gravagna refers to a similar instance in 1821, when an infected vessel arrived from Alexandria, and thirteen of its crew or passengers died of plague in the lazaretto. A Maltese who had had the disease in 1813, believed himself invulnerable and volunteered to be a nurse, but after eight days he had symptoms of plague with bubo. He recovered, and, as in 1841, the disease was confined to the lazaretto. We could adduce other instances in which plague has been confined within other lazarettos, but consider the above instances amply sufficient to prove the fact of importation. They also bear on our second test for contagion, persons brought into direct contact with the sick, and suffering in a greater proportion than the rest of the population, indeed the only ones who suffered. Let us now see how far this argument can be carried out during an epidemic prevalence of plague, observing how the proportion of the attendants varies with the extent of their communication with the sick.

Now, Clot-Bey himself allows that many medical attendants died in the hospitals of Cairo; "many employées, but especially those who are most in connexion with the patients." (p. 8.) Dr. Williams (p. 284) adduces some strong confirmatory facts. He says, "The French army on first taking possession of Egypt lost no less than eighty medical officers by the plague, an immense proportion compared with that of the army generally......At length the French resorted to the expedient of employing Turkish barbers to dress carbuncles, buboes, and blisters, as well as to bleed the plague patients; and after the adoption of this measure only twelve medical officers died in twice the former space of time...... In the English army of 7883 Europeans and native Sepoys only 165 died of plague, or about 1 in 48, yet of 13 medical officers 7 died of this disease, or more than half." These are striking facts, and we shall notice others when speaking of the results of segregation, as we are about to do in tracing its preservative influence to those who are separated from diseased persons.

On this part of our subject we cannot have a better field of inquiry than Malta, where the origin of the disease was evidently not local, and the inhabitants of different districts differ so little from each other as to the circumstances of their soil, climate, or customs. In this island, during the plague of 1813, when out of a population of about 90,000, 4486 deaths took place between April and November, we find that a whole city, "shut up," enjoyed perfect immunity. The annexed sketch gives a bird's-eye view of Valetta and the smaller cities to the south of the harbour. The distance from the point of Isola to Valetta is only 360 yards. This town differs in no respect from its neighbour, Vittorioso; both are surrounded by the sea on three sides and have strong fortifications on the land side. The plague was raging in Vittorioso; it was raging in Cospiena, immediately beneath the walls fortifying the land side of Isola; but not one single case occurred in this large city (Isola) during the whole period of the epidemic. The place was the residence of many old masters of merchant vessels, who had been engaged in the Levant trade and therefore accustomed to plague precautions; and they with the assistance of their townsmen, without government aid, completely severed their city from the rest of the island, admitting no person or thing without expurgation, enforcing the most rigid quarantine, and carefully guarding the place night and day. Now this town enjoyed a *total* exemption from the plague; a monument to the Virgin commemorative of this fact stands in the central square to this day, and an annual religious ceremony is regularly observed. Here is an instance then of plague kept *out* of a town by means of separation: the same epidemic furnishes us with another instance in which it was kept *within* another by similar means. The village of Curmi, situated at the head of the grand harbour, suffered severely from the plague; and when the disease was on the decline in Valetta, Sir Thomas Maitland had this

VALETTA AND ITS ENVIRONS.

A Valetta.
B Ricasoli. D Vittorioso. F Isola.
C Bighi. E Cospiena. G Casal Curmi.

village surrounded by a wall and a series of sentinels. This cordon became gradually contracted, leaving part of the village free from restriction; and in this part alone was there plague in Malta when Sir Thomas Maitland admitted the rest of the island to free intercourse. He publicly proclaimed the plague extinct when he had shut it up within these lines, while the disease was raging within a mile of his capital; and no fresh case of plague afterwards took place. Here, then, is plague surrounded in a village of a small island, the rest of the inhabitants of that island following their usual avocations, and not one receiving the disease. Another remarkable case, showing the effects of segregation, occurred in the convent of St. Augustine, which had been in strict quarantine until a servant purchased some old clothes from an infected district, and was taken ill. A monk who volunteered to attend him was placed with him in a separate apartment, and these two died, but no other individual within the walls. At Moscow in 1770-1, the Foundling Hospital "shut up," and the inmates, 1400 in number, were completely secluded. Some workmen who got over "the fences in the night time were the only persons attacked, and these being immediately separated the contagion did not spread, although more than 100,000 persons fell victims to this pestilence in other parts of the city." (Williams, p. 282.) At Marseilles in 1720, the religious houses which "shut up" were exempt; the Bishop certified that "the plague has not penetrated into the religious communities which had not held any communication with persons without." (Williams, p. 282.) The Naval Hospital in Malta was strictly insulated, and the only case that occurred was in the person of the market-man, who had strayed into an infected family. The inmates of the prisons, nunneries, and convents, with the exception of the case just mentioned, had an entire immunity; and the proportion of cases among the military was much less than among the remainder of the population. The Sicilian regiment, which adopted strict precautions, only lost one man, who had strayed into the town; and the other regiments which adopted similar regulations, though less strictly enforced, only lost 20 of a force of 4000, a proportion 30 times less than that of the general population.

Here then are *facts*, positive facts, established beyond the possibility of question by accurate observers and faithful recorders. We have selected from the works before us, and stated circumstances to which hundreds now living in Malta can testify; and it now becomes necessary to draw the attention of our readers to the case of the non-contagionists, and the work of their grand champion Clot-Bey, which is presented under the most imposing authority. The author had resided seventeen consecutive years in Egypt, and had been placed in the most advantageous circumstances for studying the diseases of the country and obtaining every species of documentary and official information that he wished for. He successively treats of the description of the disease, its incubation, symptomatology, morbid anatomy, and treatment. Then follow disquisitions on its origin, etiology, contagionality, and lastly, on the means of guarding against its attacks, with an inquiry into the sanatory system. The aim of the work is to prove that the plague is endemic throughout the Levant; that it is never propagated by contagion nor by infection; that it is developed solely under the influence of the causes which produced it, and that it disappears with them; that its primary origin is lost in the darkness of past ages; that all therapeutic means are almost useless; that no cause assigned by any previous writer is sufficient to explain the development of the disease, and that the sole valid or necessary causes are meteorological circumstances or atmospheric conditions which he calls *pestilential constitution*, but the nature of which he does not define; that the plague is not contagious, and that therefore all quarantine institutions are useless. In connexion with this conclusion it must not be forgotten that Clot-Bey, as an officer of Mehemet Ali, must have been greatly biassed by his wish to remove the restrictions which all nations have placed upon their intercourse with Egypt; indeed he acknowledges that he commenced his observations with such a *hope*. His arguments, and those advanced by Dr. Davy, Mr. Holroyd, and Dr. M'Lean and Dr. Mitchell, among those gentlemen examined before the parliamentary committee, are simply that the plague is generated in Egypt and some neighbouring parts of the Levant by unknown local causes, being constantly endemic and often epidemic among the inhabitants; that, like other epidemics, it has a period of commencement, of maximum intensity, and of decline; that it has a regular season, never beginning before December or after March and always ceasing in June, the people on St. John's day (24th June) regularly holding a *festa* to celebrate its cessation; that this epidemic season varies in Syria and Constantinople, its annual period being later; that Persia, though yearly surrounded by plague, seldom suffers from it, and that 70 or 80,000 pilgrims depart yearly for Mecca, taking merchandize from infected places, and the disease very seldom spreads; that the clothes of many thousand persons who have died of plague are publicly sold in the market-places after St. John's day, but no accident happens to those who wear them; that the plague has appeared in houses or districts where the strictest quarantine had been observed; that numbers in free contact with plague-patients are unaffected by the disease, and that inoculation has been practised with impunity.

Let us examine each of these arguments in detail; fully admitting, as we do, the correctness of the facts just stated, but demurring at the conclusions attempted to be drawn from them. These writers believe that if they prove the local origin of plague, if they prove it to be an endemic and epidemic disease, that they establish at once a proof of its non-contagionality. But does either experience or analogy prove that diseases arising in one mode may not be propagated in another? Have we not daily proof that when the human body has received a poison, that poison is reproduced and discharged in the exhalations? The twentieth part of a drop of variolous matter introduced beneath the cuticle is converted by the vital actions into a quantity of similar matter sufficient to cover the whole surface of the body with pustules, and healthy persons exposed to the exhalations of the sufferer are subject to the same successive effects. Is it at all unlikely that in the same way the animal body receiving the plague-poison from some exhalation peculiar to certain countries, not only suffers from the specific effects of this poison, but also, regenerating and exhaling it, becomes an irradiating focus of the disease? The periodical commencement and decline of the epidemic is also used as a strong argument against contagion, for it may be said, if it spread by contagion from May to June, why not from July to August and so on, keeping up a perpetual series of cases? But this may only show that the energy or intensity of the cause varies with the season; that the terrestrial emanations or atmospherical impregnations which produce the plague are only formed at certain seasons. We have never a perpetual series of cases of smallpox in any district, of typhus, scarlatina, nor measles. These diseases arise, arrive at a certain maximum of intensity, and then decline. The epidemic has ceased with the cessation of the atmospheric or terrestrial cause, and then no cases are met with but such as arise from direct contagion, which become more and more rare and the disease is at an end. Some morbid matter is thrown into the atmosphere and the disease commences as an epidemic; it is reproduced in the bodies of the infected and spreads by contagion while the atmospheric cause is still in operation, and this is the period of maximum intensity. Then when the epidemic cause ceases the disease declines, its propagation being effected by contagion alone.

It may be said, and said truly, that sporadic cases of plague prevail throughout the year in Lower Egypt, and if the disease be contagious, why does it not spread? It might as well be asked why does not the physical cause engendered in that district extend its operations? Or how is it that in the same district the number of cases is very small, while all the inhabitants are exposed to its operation? The fact is, that the cause of the periodically increased intensity of plague, like that of the fevers of the West Indies, of the Guinea coast, and of Ceylon; the variola of some parts of Africa; the yaws of Guinea; and the cholera of Hindostan —is perfectly inappreciable by us. The peculiar limitation of these diseases to certain districts shows that in some of the operations of nature, the morbific matter is engendered, and that in certain seasons, or certain conditions of the atmosphere, its intensity or quantity varies, and the effects, from being merely local, become epidemic. But it does not follow that because this disease is epidemic, therefore it is not contagious, or, in the words of Dr. M'Lean, (Parliamentary Evidence, pp. 96,) "that the laws of epidemic and those of contagious diseases are not only different, but incompatible." We have just shown the fallacy of this argument from analogy with variola; it is shown in the different course of a disease purely epidemic, and that of an epidemic disease which under certain states of the atmosphere extends its ravages by contagion. The different course of the plague of Malta, in 1813, and that of cholera in 1837 was most striking. The cholera originating in the east had advanced steadily in a westward course through the north of Europe, until it reached England, when like the stream of a river diverted from its course, it turned southwards through France and Italy, and then proceeded in a south-westerly direction through Sicily to Malta. The course of the epidemic was obvious; it broke out in Malta within forty-eight hours from its appearance in Palermo; it attacked Gozo at the same time; it attacked the shipping in port; it proceeded in a certain tract, instances having been observed in which one side of a street suffered, while the other was perfectly free; the military and those who observed quarantine were attacked in a proportionate ratio with the rest of the population; and at the time of its cessation the whole population were convinced that it was non-contagious, although at the commencement all the efforts which had been made to convince them of this had been ineffectual. Now, with plague, we have seen the proofs of direct importation; and Sicily which observed strict quarantine escaped, as did Gozo until the next year, when the plague entered in a manner which we shall presently describe; the shipping, which observed quarantine, escaped from the plague; the plague did not advance in a direct line, but from one corner of the city to another, the means of its communication being traceable; we have seen that in plague the military and those who observed quarantine did not suffer in proportion with the rest of the population; and lastly from the experience they had had of plague the whole mass of the population, educated and uneducated, natives and foreigners, medical and non-medical, became universally convinced that it was contagious.

The commencement and cessation of plague at certain seasons is one of the most obscure of the laws of the disease, but proves nothing against its contagionability. This, with the facts that it "has never been known to pass the first cataracts," (Williams, p. 268;) that Persia enjoys an immunity almost perfect; and that the disease so seldom spreads during the pilgrimages to Mecca, only show that certain conditions of the atmosphere are necessary to allow of the extensive operation of the poison. Mr. Green who had resided several years in plague countries gave evidence before the Parliamentary Committee, (Report, p. 35,) that in June, about the time the plague ceases in Alexandria, "the sun has such power, that it occasions strong exhalations; a strong fall of dew, almost like rain." "I had a letter from Mr. Morier, consul-general, dated I think, in February; in which he stated, that the plague that had begun to be very prevalent, had all on a sudden entirely ceased; and that he could not account for it, unless it had been occasioned by the extraordinary continuance of dense heavy fogs." At Smyrna it generally ceases in the month of July when the falls of dew are very heavy. This law of periodical cessation is only observed in countries in which the plague may be considered as indigenous, its continuance having been very variable in Malta, Marseilles, Moscow, &c. And what does it prove with regard to the question of contagion, but that certain atmospheric conditions are necessary either to predispose persons to the action of the poison, or even to admit of the existence of such a poison? just as certain conditions of air, water, and temperature are necessary to the process of fermentation. These conditions may all be present, but in the absence of the fermenting principle, no fermentation follows, and on the other hand without these conditions, the fermenting principle is inert.

We shall refer to the argument of the immunity of those wearing the clothes of dead plague-patients after St. John's day, when speaking of the question of contagion by *fomites*. As to the fact that plague has appeared in houses or districts where strict quarantine has been observed, it merely proves, what we have freely admitted, that in certain countries the specific causes exists in the atmosphere. All the facts on which this argument rests were observed in plague countries, and it is singular that no such facts have been observed in countries into which the disease has been imported, as Malta or Marseilles, where, as we have seen, the results of segregation were most conclusive. The results of inoculation we have shown to be contradictory, but certainly as much in favour of the contagionists as against them. With regard to the fact that numbers in free contact with infected persons do not take the disease, a little examination is necessary, owing to a remarkable instance which lately occurred on the coast of Syria, and which was made the subject of a communication from Sir W. Burnett to the Lords of the Admiralty. It appears that in December, 1840, the Zebra was driven ashore at Kaiffa, and some part of the crew were employed to repair the fortifications of St. Jean d'Acre, where several cases of plague had appeared among the inhabitants. On the 17th of February, the Castor arrived at Kaiffa, and on the 20th, fourteen men belonging to the Zebra were sent on board the Castor in exchange for a party of artificers, the working party employed at Acre having returned to the Zebra five days previously. On the evening of this day, the 20th, one of this party was taken ill and died on the 22d, of plague, and between this day and the 25th, thirteen more cases were added, who were immediately embarked on board the Castor, which sailed the next day for Malta. The disease proved to be most unequivocally the plague; and the remarkable point of the affair is that the only man belonging to the Castor who was attacked by the disease, was one of those who had been employed on shore, and lived with the Zebra's men at Kaiffa. The strictest precautions were taken to separate the infected persons from the rest of the crew of the Castor, and eleven men were selected solely to attend upon them, but none of these men, neither of the medical officers, indeed none but the men who had been on shore took the disease. It must be noticed, however, that great care was taken by the eleven men and by the medical officers, to effect the freest possible ventilation, and avoid actual contact as much as possible, and washing carefully after dressing the buboes. It is singular also that only one of the party of artificers sent on shore from the Castor, should have taken the disease. Dr. Johnson, in the 70th number of the Medico-chirurgical

Review, p. 599, speaking of these facts, says, "if anything stronger than this document can be necessary to prove the idle fears of contagionists, it must be a sign in the sky, like that which Constantine shaped his course by." But remarkable as it is, it is a mere negative fact, valuable in showing that under certain circumstances, plague may not be necessarily contagious, that predisposing causes may have great influence over its progress, and that free ventilation, especially of sea air, may obviate in a great measure the danger even of direct contact. It proves nothing against positive facts, in which contagion has been traced, any more than the fact of a man having cohabited with impunity with a diseased female, could prove that syphilis was not contagious. Yet examples of this circumstance are frequently met with, it being by no means uncommon for two persons to have connexion with the same female, the one receiving the disease, the other escaping. Persons may attend upon smallpox patients without becoming infected, and no one considers this a proof that the smallpox is not contagious, but that the parties were not predisposed to the disease at the time. Now that in the case of the men of the Zebra, there were predisposing causes for disease is evident, for they had been wrecked, consequently obliged to work hard, and submit to many privations, besides being lodged in a place "which is described as being most filthy with all kinds of abominations." Why only one of those men of the Castor sent on shore should have taken the disease is not the least curious part of the affair. That there is something in sea air which tends to check the progress of plague appears very probable from the evidence of Mr. Green, (Parliamentary Report, p. 37.) He states that he believes that there is no instance on record of any English sailor dying of the plague on board British merchant ships in Turkey, although he has taken pains to investigate this point. He attributes this to their different habits, and also to their "in general sleeping on board their ships, where there is a great difference in the atmosphere, from what it is on shore, perhaps eight or ten degrees." As in other contagious diseases, a sort of concentration of the poison appears to be necessary to ensure the propagation of plague; thus there is but little danger in approaching a patient labouring under any disease in a large open well-ventilated apartment, but crowd several into one room, or leave one in a small confined chamber, so that the pulmonary and cutaneous secretions and exhalations are collected into narrow space, and enter the lungs of the attendants in this concentrated state, in all probability the disease will be excited unless previous attacks or peculiar circumstances have exhausted or destroyed the susceptibility of the party. Dr. Marcet once collected a number of cases of typhus into one of the wards of Guy's Hospital, for the purposes of clinical instruction, when all at once the disease began to spread to other patients and to the nurses, although it had not done so when the cases were distributed throughout the hospital, and it ceased to do so when they were again divided. Whether the free ventilation, then the sea air, the previous healthy condition, or the short exposure to the "abominations" of Kaiffa of the men of the Castor, may account for their escape we know not, but must insist that if well-authenticated proofs of contagion can be adduced, no negative fact can controvert them.

It is with regard to actual contact being necessary for the propagation of plague, as the majority of contagionists believe and as it was firmly believed by those who witnessed the plague in Malta, that the argument has been carried too far; because the proofs of local origin and extension in certain districts independent of personal contact are indisputable, and all the facts brought forward in support of this view merely prove that the infecting distance is small. It seems highly improbable that any exhalation should be formed upon the skin of a plague-patient so powerful in its effects, that applied to the cuticle of the fingers (not a highly-absorbing surface) it should propagate the disease; while the pulmonary exhalations, the very *excretions of the blood,* applied to the highly-absorbent surfaces of the air-passages, should be innocuous. It must be proved that the morbid poison is not volatile, that it is fixed like that of syphilis and hydrophobia, before such an improbable assumption can be received. The effects of segregation in Malta, in which the distance between the diseased and those separated from them was very small, do not prove the necessity for actual contact, but simply that the exhalations are not infectious at any considerable distance, a certain dilution with atmospheric air appearing to render them innocuous.

We have thus given the sum of the information to be derived from the works before us, and the results of our own inquiries in the Mediterranean, with regard to the means by which plague is propagated. We have now to consider the bearing which the knowledge we possess of the laws of the disease has upon quarantine regulations; how far these regulations are capable of arresting its progress, and in what respects they are defective or injurious to the common good of society.

II. The conclusions on which all quarantine regulations are founded are simply these: first, that the poison of plague and of some other contagious diseases can lie dormant in the body for a certain period, and *only* for a certain period; in other words, that the poison has a definite period of latency; and, secondly, that various articles of merchandise, wearing-apparel, &c., are capable of containing the contagious principle and of conveying it in sufficient intensity to propagate the disease, while some other articles have not these properties. Classes of susceptible and non-susceptible substances are thus formed, and the former are supposed to vary in their degree of susceptibility. The object of the regulations is to prevent communication for a definite period with persons, ships, and goods arriving from certain districts, and to purify all clothes or other articles supposed to be capable of absorbing and retaining the contagious principle. These purposes are effected by means of lazarettos, which are establishments in the vicinity of an anchorage, provided with apartments properly constructed for separating persons who have different periods of quarantine; a pest-house for plague patients, warehouses for the deposit of goods brought from suspected ports, and every convenience for their purification. The duration of the quarantine varies according to the port from which the ship arrived and the nature of her bill of health. This bill of health is a certificate given to the commander of all vessels at the date of their departure from any port supposed to be subject to contagious diseases, or any port in free communication with such suspected places. It is signed by the consuls or other constituted authorities of the port, attesting the state of health of the locality at the time of the sailing of the ship. These bills are said to be "clean," "suspected," or "foul." The first imports that no infectious disease was known to exist; the second, that there were rumours of such disease, but it was not known for certainty; the third, or the absence of a clean bill, shows that the port was the seat of some epidemic or contagious disease.

Before speaking of the efficiency of these regulations, their inconsistencies or abuses, it is necessary to make some inquiry with regard to the latency of plague, and the fact of its contagion by fomites, as grounds on which the regulations are founded.

1. Now, in making inquiries with regard to the exact period of latency of plague, it is necessary to lay aside all evidence which has been collected in situations exposed to epidemic influence; because, in this case, if the disease show itself within a given period after exposure to contagion it is by no means certain that the exposure and the disease stand in the relation of cause and effect. This objection removes almost all value from the observations of Bulard, Clot-Bey, and others in plague countries. Some of the remarks of M. Ségur-Dupeyron are of considerable value. He says (Rapport, pp. 26-33) he had noticed that the cases of plague which had occurred in the various lazaretti of Europe since 1720 "had all been conveyed and communicated by men; and that, with two or three exceptions, the disease showed itself during the voyage." M. Dupeyron extended his researches to the East, and by the aid of consular authorities furnished a report in which he shows that "in 65 cases of maritime importation which it contains, the disease has broken out on board of 50 vessels during the voyage." Unfortunately he has not given returns of the actual length of the voyages, or cited cases upon which we can rely in determining the exact period of latency; but states as the result of his inquiries that 11 days may be considered as the extreme period. Dr. Aubert, in a memoir read before the Academy of Sciences in Paris, September 1841, states that in the space of 124 years, 64 vessels having plague on board have arrived in the lazaretti of Europe, and that in 26 only has the disease continued after the arrival; that it has never shown itself after the arrival unless it had previously appeared during the passage; and that the period of latency on board has never exceeded eight days from the date of departure. At Malta in 1813 the disease was said to show itself *generally* from three to six days after communication. Dr. Williams quotes from Aubert the fact, that "four criminals condemned to death were inoculated for the plague, and they all laboured under the disease before the fifth day." (Williams, p. 298.) Clot-Bey believes the mean period to be from three to six days, and that the extension to eight days is rare. The whole credible evidence goes to prove that the period is much shorter than is generally supposed; but more accurate information must be obtained before satisfactory conclusions can be arrived at.

2. On the question of contagion "per fomitem," the most contradictory evidence is advanced, and on a belief in such a mode of propagation not only are founded the quarantine regulations for the depurgation of merchandise, but also the duration of the term of quarantine of the passengers and crew of the vessels carrying suspected articles. It is on this belief that the quarantine authorities explain the apparent absurdity of giving steam and sailing vessels the same period of segregation. If the cargo be infected, and kept in the close hold of a ship, it matters little whether the voyage has been four or five days in a steamer or two or three weeks in a sailing vessel, because the crew must be detained under observation until such a time after the free ventilation of the cargo, as shall convince the guardians of the public health that no one has suffered from the exposure of the cargo and their contact with it. This is perfectly reasonable if mediate contagion be allowed, and it is clear that the period of quarantine should then in every case be regulated by the extreme period of latency of plague after the perfect ventilation or depurgation of the cargo ; less than this would be obviously unsafe, more unnecessary. But let us inquire what are the facts on which the believers and sceptics in this mode of propagation rely ? Bulard (pp. 49-54) narrates two cases in which condemned criminals were dressed in the shirts and drawers taken from plague-patients and placed in the beds still warm and impregnated with the perspirations of the diseased. Both took the disease and one died. Bulard submitted himself to the same experiment and experienced no ill effect, and states as the result of his experiments and inquiries "that this mode of transmission, in the actual state of science, can neither be sustained nor combated logically." (p. 49.) Sir James M'Grigor, in his evidence before the parliamentary committee, (Report, p. 60,) when asked if the plague "can be produced from goods and things," says, "I can speak with certainty of the clothing of men; and I can also speak with certainty that blankets have conveyed it." His observations were made with the army in Egypt, and the officers of the French army became so convinced that captured articles of dress communicated the disease, that Napoleon ordered all such articles to be burnt. Mr. Green, who resided many years in Constantinople states, "that he does not think clothes or goods can convey the disease, goods certainly not." He is asked (Report, p. 34,) with regard to the habits of the Turks:

> "What do they do with the clothes belonging to persons who die of the plague? Sell them; they never destroy them.
>
> "Have you ever known the clothes to be the cause of the plague in other persons? No; I have strong reason to think they are not the cause.
>
> "Why? Because the people who deal in them are not affected.........
>
> "Even the bedding is sold, you say? Even the bedding is sold. There is a custom in Turkey, that if a stranger dies in the plague, the Governor or Vasha takes possession of his property, and the clothes are part of the property; and of course he orders them to be sold for his own benefit, and they dare not destroy them."

M. Dupeyron (Rapport, p. 112,) records a curious fact which bears both upon the duration of the period of latency and the infecting power of merchandise. "A ship commanded by Captain Maranguvich arrived at Venice in 1818. This ship was submitted to thirty days of quarantine. Two days only were required to be admitted to free pratique, when a passenger named Micheli Cetti rummaged his trunk to find his purse, and immediately contracted the disease. It was afterwards discovered, that owing to the carelessness of the guard this trunk had never been opened." This is all M. Dupeyron states of this case; and if the facts be correctly stated, and that the rest of the merchandise had been purified at the time of the ship's arrival, (which is perhaps doubtful,) either a period of latency of twenty-eight days must be allowed, or the possibility of infection by mediate contact. Yet we find M. Dupeyron (p. 122) allowing "that there are no positive proofs that articles of merchandise have communicated plague in the lazarets;" and it seems to be a generally acknowledged fact that the merchandise of vessels in which plague was not actually on board, has never communicated the disease in any lazaretto. We have just alluded to the custom of selling the clothes and bedding of those dead of plague in Turkey; it would appear that this is done in Egypt and Syria with equal impunity, for Clot-Bey states that the day after St. John's day, such sales take place openly in the market-places, and without ill effect. He says that in 1836 the effects of 50,000 persons dead of plague, were sold in the bazaars without previous disinfection, and in no single instance did they communicate the disease. He adds, that upwards of 3000 patients were treated in the plague-hospital in Cairo, but that at the close of the epidemic, although through carelessness other patients were placed in the same bed, under the same woollen counterpanes, and with no other change than the blankets, yet no individual caught the plague. When we compare these facts with the experiments of M. Bulard, where the disease actually followed in two cases the exposure to fomites, it appears that disinfection of clothes and bedding is very rapid, and that the poison of plague is readily decomposed by free access of air or slight changes of temperature; and this is of great importance in its bearing on quarantine regulations, giving us some assistance in determining the period during which the contagious principle can be preserved in an active state in wearing apparel or merchandize. This period would appear to vary with the circumstances under which the fomites may be placed, as in the best authenticated cases of contagion 'per fomitem,' the suspected substance has been kept away from air and light. One most extraordinary instance occurred in Malta in 1813, which shows, at the least, that a full examination of the whole subject, and of the effects of ventilation as a disinfectant, must be made before the facts cited by Clot-Bey and Mr. Green can be considered to prove that fear of mediate contagion is groundless. We have described the state of Casal Curmi, in which the plague raged after the rest of Malta was free; in this village a man employed as a burier of the dead stole and buried in a box some articles of wearing apparel. Upwards of two months after the plague had ceased in Casal Curmi, this man dug up the box and carried it to the island of Gozo, which island had adopted the strictest quarantine, and had remained to this time perfectly free from plague. A marriage was about to take place in his family, and he opened the box to present a silk covering for the head (a *faldetta*) to the bride. He, the female, and a priest who was present, all became affected with plague from that day and died, and from that family it was clearly traced to others, and finally spread over the whole island. This fact is known to hundreds of persons now living in the islands of Malta and Gozo, and was recorded in the government despatches of Sir Thomas Maitland. It must be recollected that these islands are separated by a channel scarcely a mile in width; that their soil and productions, and the manners of the inhabitants are similar; that during the time the plague raged in Malta, Gozo entirely escaped, having adopted strict quarantine; that a man after the cessation of the disease in Malta takes wearing apparel of persons dead of plague to a relation's house, opens it, and he himself and those in contact with him die of plague, and then the disease is traced over the island; and lastly, that Malta put Gozo into a strict quarantine of forty days, and plague did not reappear in the former island.

We may conclude, then, that although there would appear to be great danger of contagion from the clothes of plague patients which have not been exposed to air and light; that the danger of contagion, by means of merchandise, or even of clothes and bedding that have been exposed to the air, is by no means established, and we may add, without entering into the subject, that the present distinction between susceptible and nonsusceptible articles, does not appear to have any foundation in scientific principles. We think we have shown that plague is not a simply *contagious* disease, like syphilis, vaccinia, or hydrophobia; that it is not a simply *infectious* disease, like the remittent and intermittent marsh fevers; but that, like variola, it is both contagious and infectious, although the infecting distance is but small. The definite period of latency is as yet undetermined; but the whole credible evidence would prove that it has never exceeded fifteen days, probably has always been within ten. It necessarily follows that the belief on which the whole quarantine of the Mediterranean is carried on, that the disease is simply contagious and admitting everything but actual contact between healthy and suspected individuals, is unfounded, and the system based on it must be unsound in principle and faulty in operation; and the regulations of the different sanatory bodies not having fixed principles to guide them in regulating the duration of quarantine, and therefore being led by individual opinion, must give rise to the most glaring inconsistencies. Thus two travellers leave Constantinople on the same day; the one passes by land through Austria, and arrives in England in free pratique a fortnight before the other, who has come by sea, and who is still subjected to a long period of quarantine. Lastly, it would appear that many of the most injurious commercial restrictions, based as they are upon the fear of contagion by merchandise and upon the capricious distinctions between susceptible and nonsusceptible articles, might be removed with perfect safety to the public health, and with great advantage to society in general.

Admitting, then, that there are many glaring inconsistencies in the present quarantine regulations, and doubtless many abuses in the mode of carrying them into effect, we are still of opinion that the faults of any institution, the object of which is salutary, should lead, not to clamours for its total abolition, but to well-directed efforts towards its reformation. Let us inquire how may this be effected? and at the first glance it will appear that two objects must first be obtained: the one, a more accurate knowledge of the natural history of plague on those particulars with which we have shown our present information to be defective; the other, a proper understanding between the boards of health in the different states of Europe and in the Levant, by which a uniform and consistent period of quarantine and some harmless method of expurgation would be agreed upon. If it be asked how are we to obtain these desirable objects? we reply by a method which has been recommended by every practical man who has written on this subject; the appointment of a commission of medical men by government to examine into the whole question, and furnish the necessary scientific information on which to base all sanatory regulations.

As we have seen something of the lazarettoes in the Mediterranean, it may not be amiss to give a few hints on the formation of such a commission, and their chief points of inquiry and best mode of obtaining the requisite information. In the first place, then, we should say, let men be selected who have had opportunities of studying the subject, and who have a sufficient knowledge of French and Italian to avoid the misconception of an interpreter; and we may add that Government might find such men among the medical officers of the navy, and thus carry on the inquiry with scarcely any expense. Two or three such individuals having been selected, their first object would be to obtain the full cooperation of the board of health of Marseilles, because this port virtually governs the whole quarantine of the Mediterranean. For instance, if Malta were to admit the Barbary states to free pratique, the Marseilles authorities would at once place all vessels from Malta in fifteen days' quarantine; and the same with other ports. In fact, being the chief port of the Mediterranean commerce, her authorities have merely to say "put a certain place in the quarantine we require, or we shall put quarantine upon your port," to have their wishes complied with. Thus it is evident Marseilles is the point whence to commence operations. Then it will be necessary to make inquiries in the lazarettoes of Genoa, Leghorn, Naples, Malta, Venice, Sicily, &c. with regard to the cases of plague which are recorded to have occurred in these lazarettoes, the places from whence they were imported, the state of health in that place at the time, and the length of the voyage, in order to determine as nearly as possible the period of latency in every case. With these inquiries will of course be combined others to determine if the fear of contagion by merchandise has any foundation in experience; whether any scientific classification can be made of susceptible or nonsusceptible substances; and whether living animals, not being subject to the plague, can convey it. If clothes or merchandise be found capable of conveying the disease, the most simple, speedy, and economical mode of depuration must be determined. There are many other points which will suggest themselves to any one who has read the present article. The commission extending its researches into the Levant, more accurate knowledge might certainly be obtained of some of the laws of the disease, and its relation with other endemic fevers of the country. It appears extremely probable that more accurate information might be gained with regard to the *origin* of the plague, and that though this is confined to Lower Egypt, it may arise from some habits of the inhabitants of the Delta, particularly that of throwing the dead bodies of animals into the Nile which decompose upon its banks in large numbers. Be this as it may, it is now universally observed that whenever there is much sickness among the cattle, a plague season is sure to follow, and perhaps this and other habits of the people may be as much the cause of plague as our old prison discipline was the cause of gaol fever. The plague was formerly prevalent from time to time in Europe; since the establishment of sanatory laws, this has become very rare, and then, as in the case of Malta, it could be traced to their infringement. The uninterrupted existence of the plague in the

Levant for a long series of years led to the belief that it was indigenous in the whole Ottoman empire; but after the occupation of some Turkish provinces by Russia, and the establishment of proper sanatory regulations, these provinces were kept as completely free from plague as the rest of Europe. This belief was again shaken, when, after the introduction of sanatory laws into the new kingdom of Greece, that country was completely emancipated from the scourge. Latterly, the rules of quarantine have been observed in Turkey, in Smyrna, in many parts of Asia Minor, in Syria, and in the Barbary states, and since their establishment plague has not made its appearance, Constantinople having been perfectly free from plague for upwards of five years, although formerly it was so universally present, that the authorities laughed at sanatory rules, and believed that a focus of plague existed in every corner of the city. May we not hope that in Egypt a proper system of drainage, burial of dead animals, and improvement in the dwellings and customs of the people, with proper separation of every case of plague as it arises, may gradually extirpate the disease, as they have done in Turkey? These plans Mehemet Ali has every disposition to follow, and ample means of carrying into effect; and if properly followed up it is not impossible that we might see Egypt as clear of plague as our own gaols are of typhus, or, to say the least, as our own marshy districts, or districts which were marshy, are of ague. At any rate let the inquiry be made, putting aside all theory, and disinterestedly searching for the truth from disinterested persons; and then we shall probably see a modern crusade against an exterminator of the human race which our own ignorance and evil habits have created.

Before concluding we may add that we have made many of the inquiries above recommended, and may therefore speak on something more than supposition as to their probable beneficial result. We believe it will appear that after an experience of 200 years the plague has never been brought into any lazaret except by ships coming from places where it was raging at the time of their departure; and that no well-authenticated case has been observed where the period of latency has exceeded ten days. Why then subject whole countries to quarantine where the plague is never engendered, and which take proper precautions against those places where it does reign? And why continue the system of twenty and thirty days' quarantine? If a *foul* bill of health were required only from places where the plague prevailed; and a *suspected* bill only from ports in free communication with infected districts; while ships from all places which take what may be agreed upon as the necessary precautions, should be received with clean bills, and in free pratique, every security would be given to the public health and immense advantages result to society. The whole of Barbary, Syria, Greece, the Ionian Islands and Candia would be thrown open to the free commerce of the world, and a great advance thereby gained in civilization and the promotion of human happiness. Barbary, for example, by means of Malta, would be brought within a day's journey of the centre of the civilized world, into free communication with a people speaking the same language, enjoying the benefit of liberal institutions, and anxious to establish commercial and friendly relations, which are now rendered almost impossible by the expense and delay of fifteen days' quarantine, which all experience has shown to be perfectly useless. The Ionian Islands, Greece, and Syria, again, being brought into free communication with each other, mutual good offices are exchanged, the dependence of one state upon another made clear to all, selfish prejudices wiped away, and many obstacles removed from the attainment of the philanthropic desire to reduce man into one great family; a great moral empire in which reason, intelligence, and social improvement take the place of despotism, ignorance, and the sword.

Some may think that by such measures the public health would be endangered; we reply, on the reverse, it would be rendered much more secure, because the quarantine regulations upon vessels really liable to import the disease would be much more strictly and securely carried into effect than is at present possible. Even in Malta, where they have more space than in any other quarantine harbour in the world, it is evident to all that a hundred vessels in different periods of quarantine must be more or less crowded, and although each has a guardian on board, and some classification of the ships is adopted, yet it is impossible to be certain that communication may not at all times take place between them. This must be much more liable to occur in the confined harbours of Marseilles, Genoa, and Leghorn, and the public health is thus far more endangered than it would be by admitting ships from the places we have named to free pratique, and leaving those from districts really dangerous under the full observation and complete segregation which their small number would render easy and certain.

We have taken up this subject at a greater length than we intended: but we are deeply impressed with its importance, as one in which the medical profession must necessarily be the guide of the legislative power; and in which, by the force of reason and intelligence it may wipe away old prejudices, advance the progress of civilization, and thus adding vastly to the general sum of human happiness, shine forth more clearly than ever, a great benefactor of the world.

---

## Figures and Tables

**Figure f1:**